# Supplementary material for: Integrative proteogenomic characterization of hepatocellular carcinoma across etiologies and stages
Source: Nat Commun. 2022 May 4;13:2436. doi: 10.1038/s41467-022-29960-8 (PMC9068765; doi:10.1038/s41467-022-29960-8)
Supplement: Supplementary file 1 — Supplementary Information [file 41467_2022_29960_MOESM1_ESM.pdf]

Proteogenomic characterization of hepatocellular carcinoma

Ng et al.

Supplementary information

Supplementary Figure 1

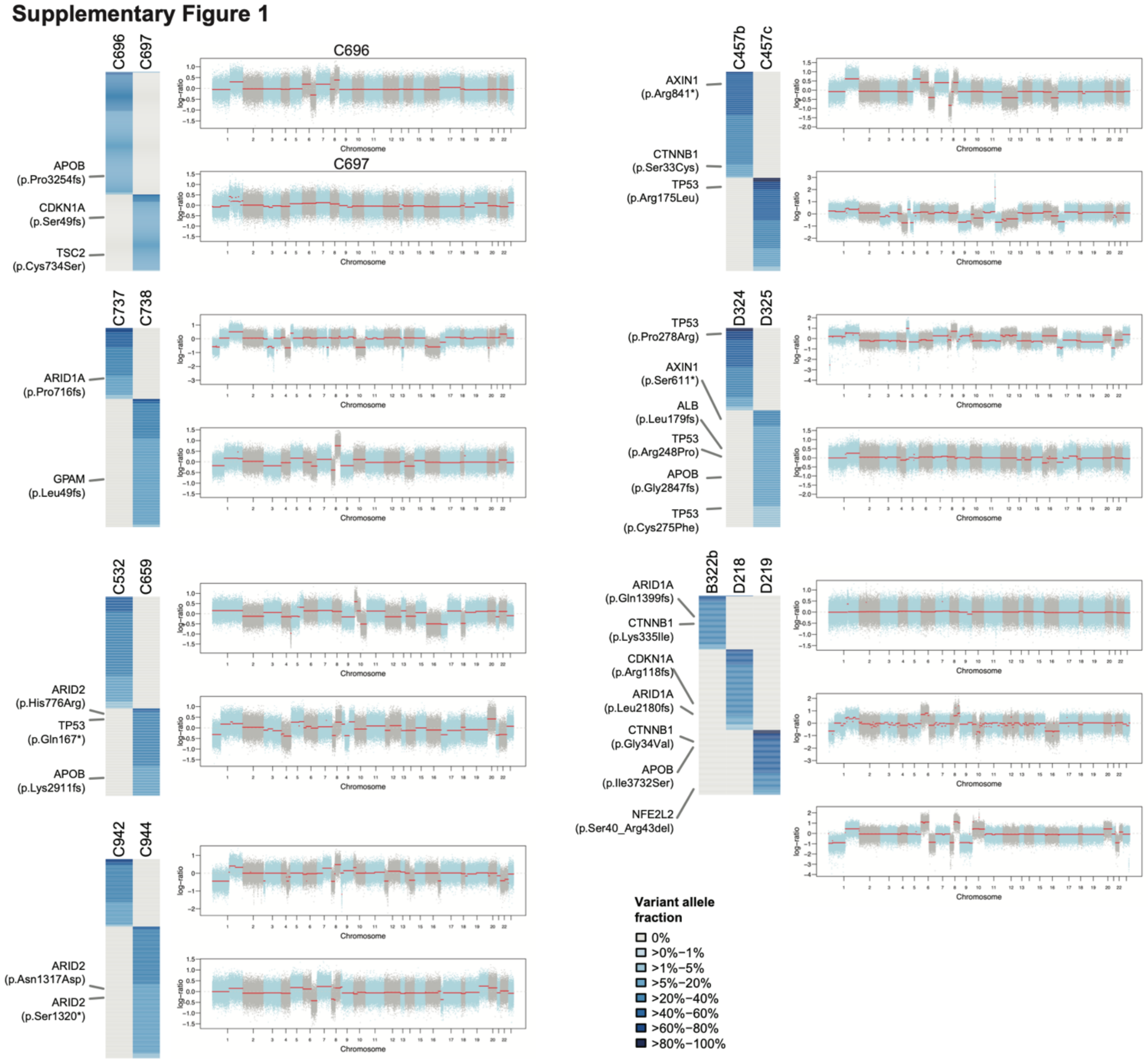

Supplementary Figure 1: Genetic profiles of genetically independent tumors from 7 patients. Heatmaps illustrate the variant allele fractions (shades of blue according to the color key, gray indicates absence) of the somatic mutations identified in the 7 patients for whom >1 tumor biopsy was included in the study. Non-synonymous mutations in HCC driver genes are labeled. To the right of the heatmaps are genome-wide copy number plots of the tumor biopsies. In the copy number plots, segmented Log2 ratios (y-axis) were plotted according to their genomic positions (x-axis). Alternating blue and gray demarcate the chromosomes.

Supplementary Figure 2

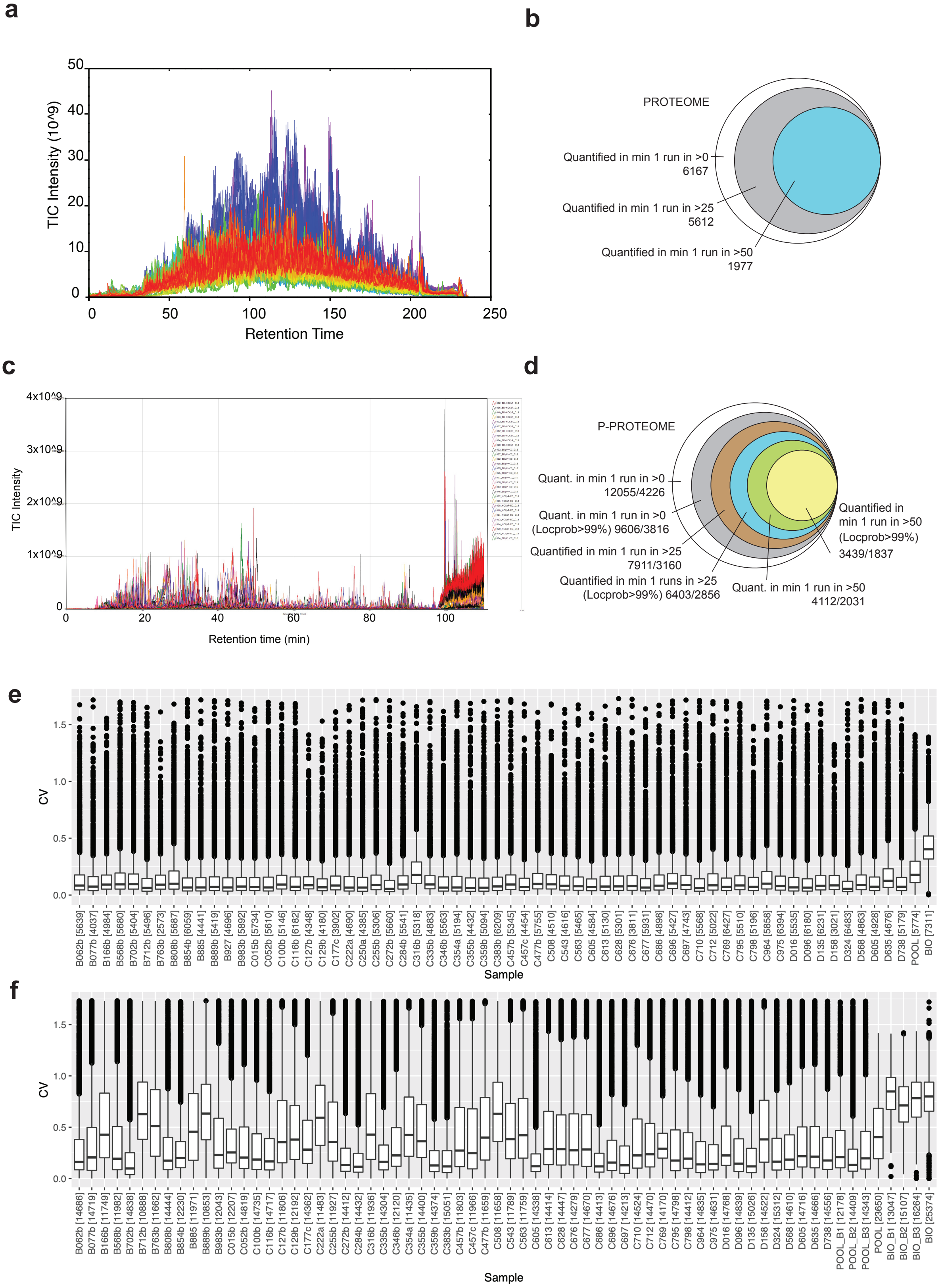

Supplementary Figure 2: Statistics of proteomics and phospho-proteomics data. (**a**) Total ion count (TIC) overlay of proteomics DIA SWATH runs of all biopsies. Panel was generated as QC panel in Spectronaut (Biognosys). (**b**) Number of proteins quantified with indicated statistics thresholds. >n where n is the number of tumors in which protein was

quantified. (**c**) Total ion count (TIC) overlay of phospho-proteomics DDA label free runs of all biopsies. Panel was generated in RawMeat from 30 randomly picked MS runs out of all biopsies. (**d**) Number of p-sites quantified with indicated statistics thresholds. Locprob = localization probability of p-site. >n where n is the number of tumors in which p-site was quantified. (**e**) Percentage of coefficient of variation (CV) of protein identification per biopsy. Each tumor biopsy is CV of the three repeat injections. n=the number of proteins quantified as indicated in the brackets. POOL is the CV of full process technical replicates of the Pool of normal liver (NL) biopsies of which multiple aliquots were measured over the course of the project. Three runs were randomly chosen and bootstrapping was implemented to avoid bias of single runs. BIO is the CV of biological replicates showing biological variability between different tumors. Three runs were randomly chosen and bootstrapping was implemented to avoid bias of single runs. Thick line in the boxplot denotes the median; box extends to the 1st and 3rd quartiles; whiskers extend to the +/-1.5 IQR of the box; dots depict the outliers. (**f**) Percentage of coefficient of variation (CV) of p-site identification per biopsy. Each tumor biopsy is CV of the three repeat injections. n=the number of p-sites quantified as indicated in the brackets. For details see (**e**).

Supplementary Figure 3

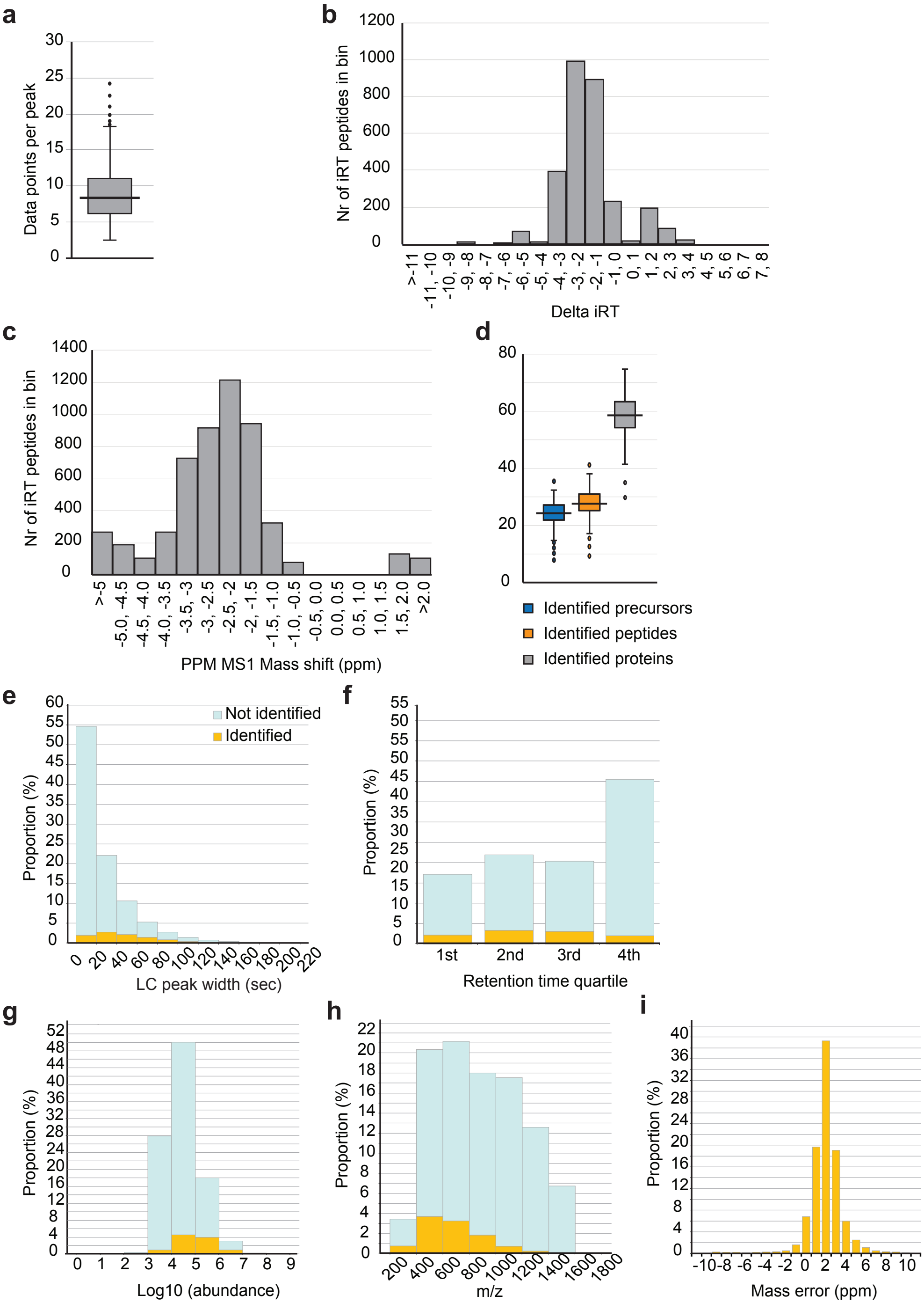

Supplementary Figure 3: Quality control of proteomics and phospho-proteomics platforms. (**a**) Data points per peak as generated from the iRT calibration kit QC panel in Spectronaut (Biognosys). Only the identified peptides from the iRT calibration kit that was added in each biopsy run are taken into account. Thick line in the boxplot denotes the median; box extends to the 1st and 3rd quartiles; whiskers extend to the +/-1.5 IQR of the box; dots depict the outliers. N = 2979 peptide measurements corresponding to 196 MS runs. These include 185 technical triplicate MS runs from 62 biopies and 11 individual MS runs from individual aliquots of the pooled control of NL. On average, 15.2 peptides were measured per MS run. (**b**) Difference of expected versus observed peptide retention time (Delta iRT) as generated from the iRT calibration kit QC panel in Spectronaut (Biognosys). Only the identified peptides from the iRT calibration kit that was added in each biopsy run

are taken into account. (**c**) MS1 mass shift in ppm as generated from the iRT calibration kit QC panel in Spectronaut (Biognosys). Only the identified peptides from the iRT calibration kit that was added in each biopsy run are taken into account. (**d**) Identified precursors, peptides and proteins as generated from the iRT calibration kit Proportion in percent (%) of the precursors/peptides/proteins from the spectral library that could be quantified as generated from the iRT calibration kit QC panel in Spectronaut (Biognosys). Only the identified peptides from the iRT calibration kit that was added in each biopsy run are taken into account. Thick line in the boxplot denote the median; box extends to the 1st and 3rd quartiles; whiskers extend to the +/-1.5 IQR of the box; dots depict the outliers. N = 196 MS runs. These include 185 technical triplicate MS runs from 62 biopsies and 11 MS runs from individual aliquots of the pooled control of NL. (**e**) Proportion in percent (%) of peptides eluting per gradient quartile as generated by the QC step in Progenesis QI (Nonlinear Dynamics). Yellow = identified peptides, blue = unidentified peptides. (**f**) Proportion in percent (%) of LC peak width in seconds (sec) as generated by the QC step in Progenesis QI (Nonlinear Dynamics). Yellow = identified peptides, blue = unidentified peptides. (**g**) Proportion in percent (%) of peptide Log_{10} abundance counted per bin as generated by the QC step in Progenesis QI (Nonlinear Dynamics). Yellow = identified peptides, blue = unidentified peptides. (**h**) Proportion in percent (%) of peptide m/z value counted per bin as generated by the QC step in Progenesis QI (Nonlinear Dynamics). Yellow = identified peptides, blue = unidentified peptides. (**i**) Proportion in percent (%) of peptide mass error in ppm counted per bin as generated by the QC step in Progenesis QI (Nonlinear Dynamics).

Supplementary Figure 4

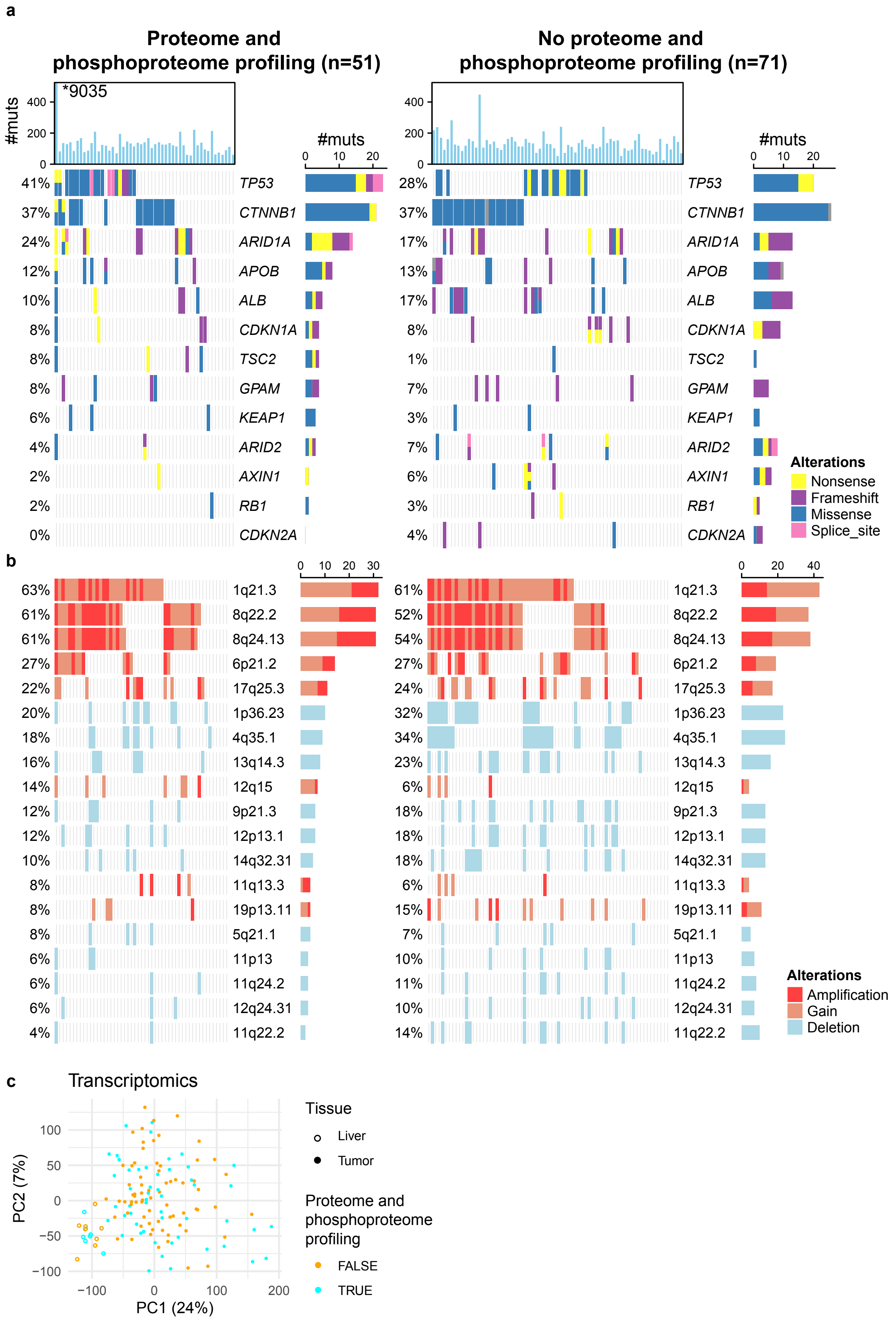

Supplementary Figure 4: Molecular profiles of HCCs subjected to and not subjected to (phospho)proteome profiling. (a) Oncoprint showing the somatic mutational landscape of HCC biopsies, stratified by the availability of proteome and phosphoproteome profiling. Significantly mutated genes in the current cohort (*CTNNB1*, *CDKN1A*, *TP53*, *ALB*, *ARID1A*, *GPAM, AXIN1)* and six additional HCC driver genes (previously reported in at least 2 studies and mutated in at least 3 biopsies in this study) are included. Barplot above the oncoprint shows the total number of somatic mutations in each biopsy. Percentages to the left of the oncoprint show the fraction of biopsies harboring somatic mutations in a given gene. Barplot to the right of the oncoprint shows the total number and type of mutations identified in a given gene. The type of mutations is color-coded according to the legend. (**b**) Oncoprint showing the copy number profiles of HCC biopsies, stratified by the availability of proteome and phosphoproteome profiling. Significantly altered regions as defined by GISTIC2 on the entire cohort are shown. Copy number status was defined by GISTIC2. (**c**) Principal component analysis plot of gene expression of HCC biopsies (filled circles) and normal liver biopsies (open circles), colored by the availability of proteome and phosphoproteome profiling.

Supplementary Figure 5

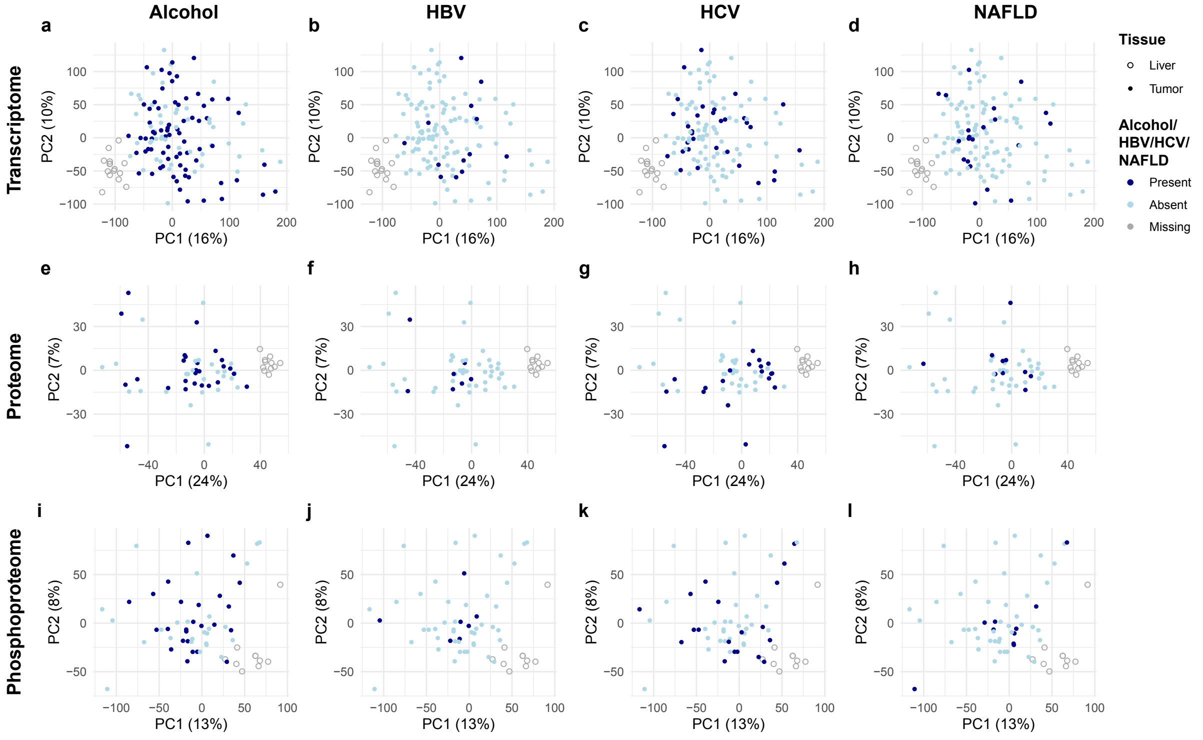

Supplementary Figure 5: Principal component analysis plots of (**a-d**) transcriptome, (**e-h**) proteome, (**i-l**) phosphoproteome of HCC biopsies, colored by the presence or absence of underlying (**a**,**e**,i) alcoholic liver disease, (**b**,**f**,**j**) HBV infection, (**c**,**g**,**k**) HCV infection or (**d**,**h**,**l**) NAFLD, and normal liver biopsies.

Supplementary Figure 6

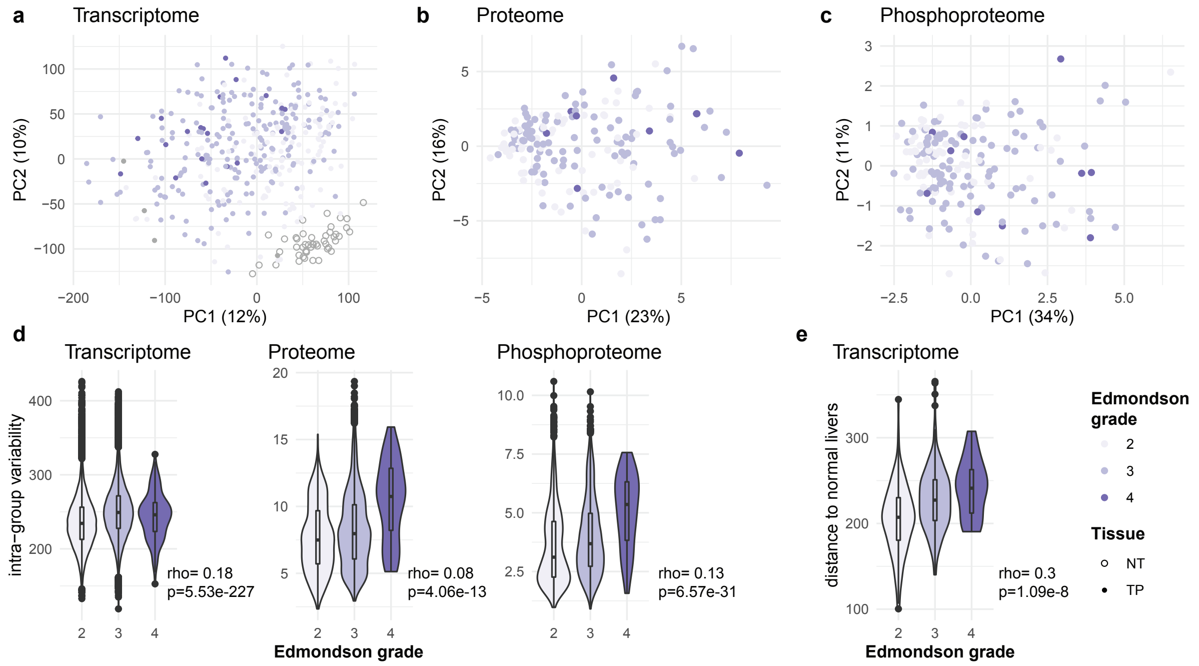

Supplementary Figure 6: (**a-c**) Principal component analysis plots of (**a**) transcriptome, (**b**) proteome, (**c**) phosphoproteome of HCCs of The Cancer Genome Atlas cohort (colored by Edmondson grade) and normal livers (transcriptome only). (**d**) Intra-group (within Edmondson grade) variability as measured by pairwise Euclidean distance between samples according to principal components. (**e**) Distance of each HCC to the median of normal livers as measured by Euclidean distance according to principal components. (**d-e**) Thick middle line in the boxplot denotes the median; box extends to the 1st and 3rd quartiles; whiskers extend to the +/-1.5 IQR of the box; dots depict the outliers. (a-e) Transcriptome: grade 2 n=123, grade 3 n=214, grade 4 n=20. Proteome and phosphoproteome: grade 2 n=63, grade 3 n=104, grade 4 n=10.

Supplementary Figure 7

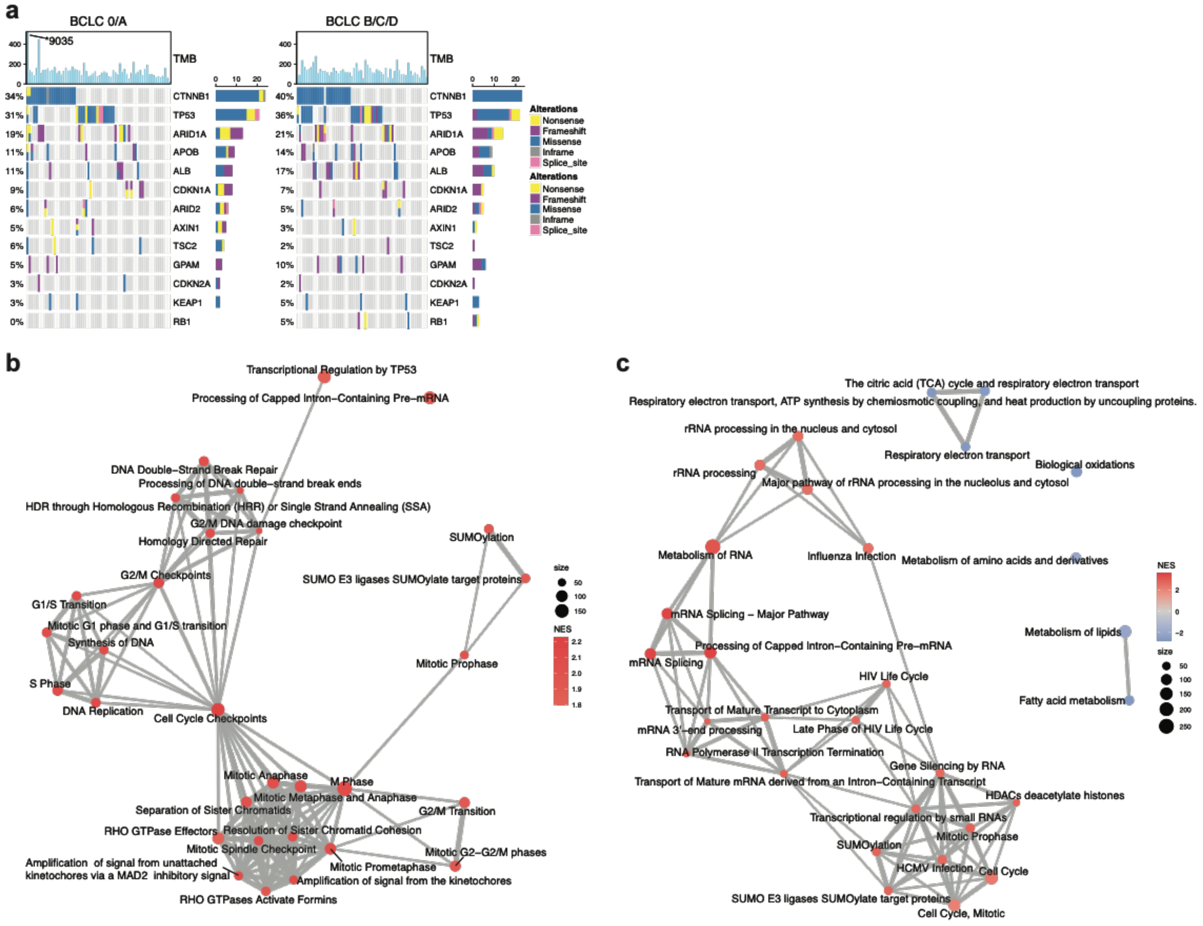

Supplementary Figure 7: Molecular profiles of BCLC 0/A vs BCLC B/C/D HCCs. (a) Oncoprint showing the somatic mutational landscape of HCC biopsies, stratified by BCLC (0/A vs B/C/D). Significantly mutated genes in the current cohort (*CTNNB1*, *CDKN1A*, *TP53*, *ALB*, *ARID1A*, *GPAM, AXIN1)* and six additional HCC driver genes (previously reported in at least 2 studies and mutated in at least 3 biopsies in this study) are included. Barplot above the oncoprint shows the total number of somatic mutations in each biopsy. Percentages to the left of the oncoprint show the fraction of biopsies harboring somatic mutations in a given gene. Barplot to the right of the oncoprint shows the total number and type of mutations identified in a given gene. The type of mutations is color-coded according to the legend. (**b-c**) Enrichment map showing the top 30 Reactome pathways enriched among (**b**) genes and (**c**) proteins differentially expressed between BCLC B/C/D vs 0/A HCCs.

Supplementary Figure 8

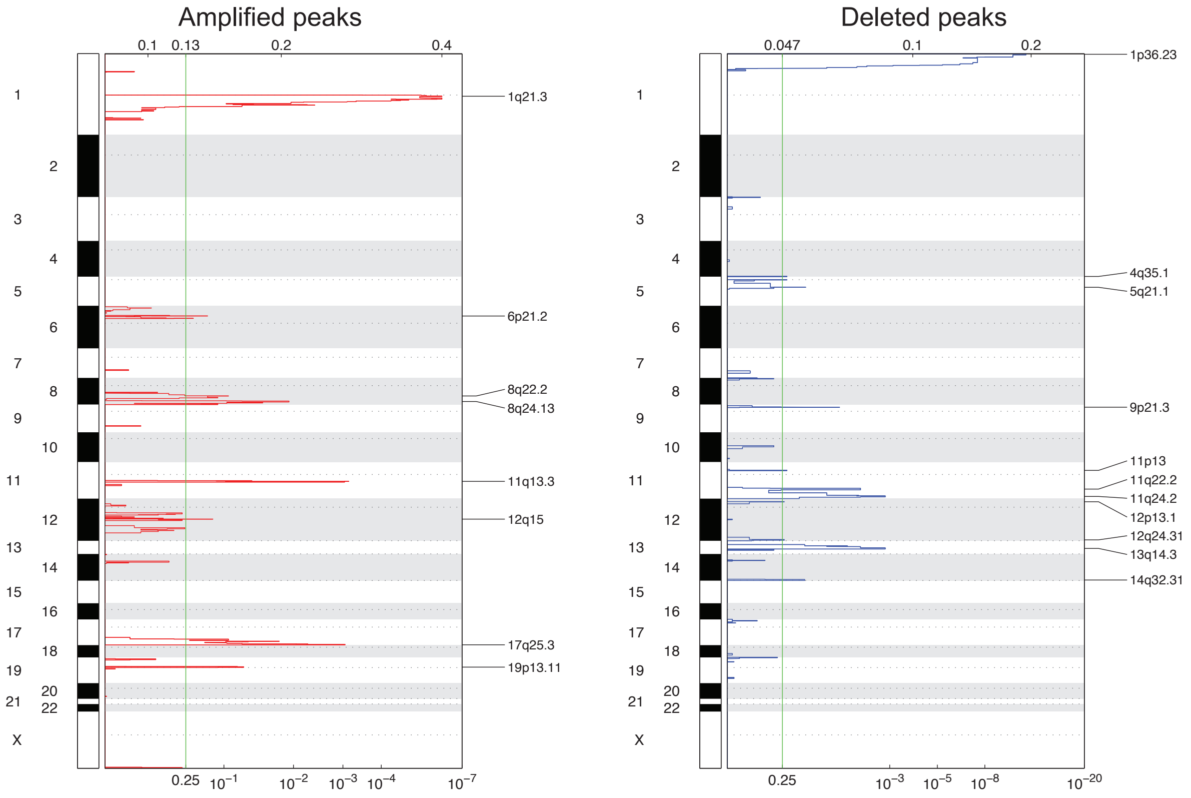

Supplementary Figure 8: Regions of the genome that are significantly amplified (left) or deleted (right) according to GISTIC2. Figures show the G-scores (top) and q-values (bottom) with respect to amplifications/deletions for all markers over the entire region analyzed. Green line indicates q=0.25 (significance threshold).

Supplementary Figure 9

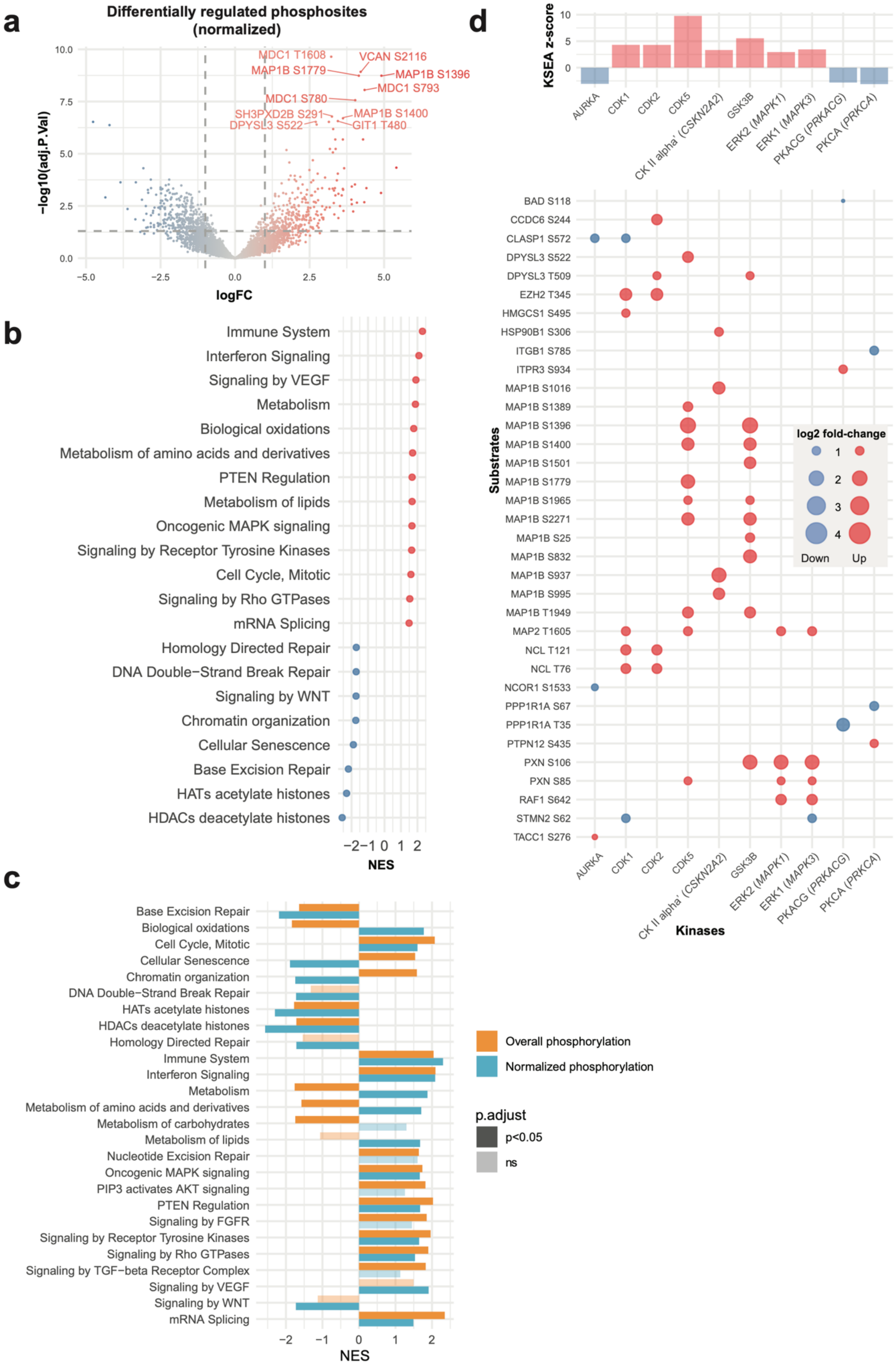

Supplementary Figure 9: (a) Volcano plot of the -log10(adjusted p-value) against the log fold-change (logFC) of the differentially regulated phosphosites normalized by overall protein levels ('normalized phosphosites') in HCC compared to normal livers. Dots are colored by logFC. Vertical dotted lines indicate |logFC|=2 and horizontal dotted lines indicate adjusted p-value=0.05. **(b)** Dot plot illustrating selected enriched Reactome pathways according to gene set enrichment analysis (GSEA) from the differential expression analysis in (**a**). NES: normalized enrichment score. **(c)** Comparison of the selected Reactome pathways according to GSEA from the differential expression analyses of overall and normalized phosphorylation. Enrichment and statistical significance were defined by gene set enrichment analysis. Multiple correction was performed using the Benjamini-Hochberg method. **(d)** Top barplot showing the enrichment z-score of the kinases with significantly up- or downregulated kinase activity in a kinase-substrate enrichment analysis (KSEA) comparing normalized phosphosites in HCC to normal livers. In the bubble plot below, the phosphosite substrates are shown in rows, where red and blue dots indicate that the phosphosite is up- and downregulated, respectively. The size of the dots is proportional to the log2fold-change of the phosphosite. Phosphosites with at least a 5-fold difference between HCCs and normal livers are shown. For kinases with <3 substrates with at least a 5-fold difference, the top three substrates with the highest |logFC| are shown.

Supplementary Figure 10

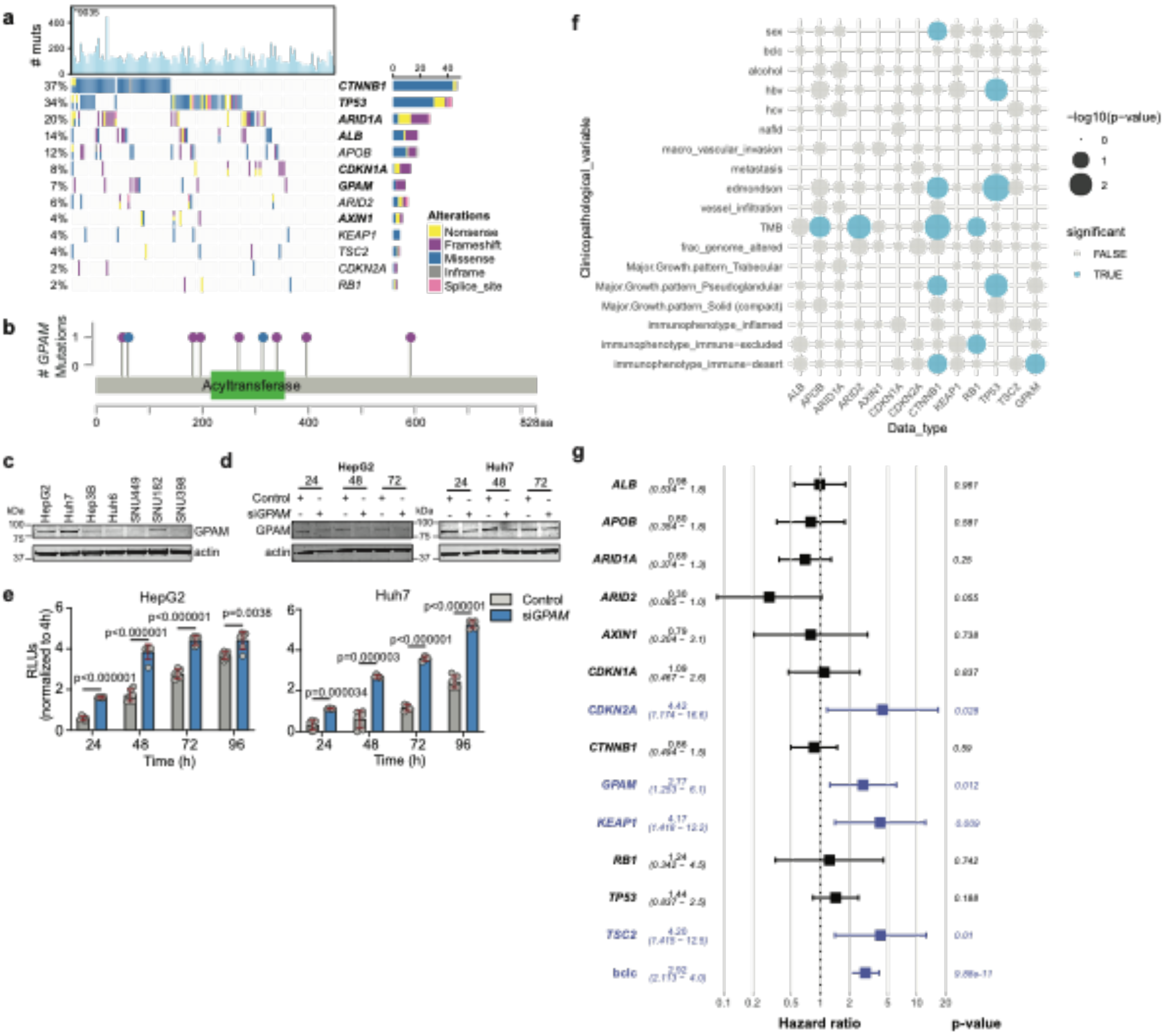

Supplementary Figure 10: (a) Oncoprint showing the somatic mutational landscape of HCC. Significantly mutated genes in the current cohort (*CTNNB1*, *CDKN1A*, *TP53*, *ALB*, *ARID1A*, *GPAM, AXIN1*, in bold*)* and six additional HCC driver genes (previously reported in at least 2 studies and mutated in at least 3 biopsies in this study) are included. Barplot above the oncoprint shows the total number of somatic mutations in each biopsy. Percentages to the left of the oncoprint show the fraction of biopsies harboring somatic mutations in a given gene. Barplot to the right of the oncoprint shows the total number and type of mutations identified in a given gene. The type of mutations is color-coded according to the legend. **(b)** Lollipop plot showing the distribution of the *GPAM* mutations along the protein, with the mutations colored according to the color key in **(a)**. **(c)** Immunoblot showing GPAM expression in a panel of liver cancer-derived cell lines. **(d)** Immunoblot showing GPAM expression in the HepG2 and Huh7 cell lines at 24, 48 and 72 h post siRNA transfection. **(e)** Proliferation kinetics of GPAM-silenced (siGPAM) cells compared to their respective controls (CTRL) normalized to 4 h after seeding. n>=5 from 2 independent experiments. Error bars show mean +/- sd. Statistical comparisons were performed by two-sided t-tests. (**f**) Bubble plot showing association between mutation status and clinicopathological parameters. Size of the circles is proportional to -log10(p-value) and blue circles indicate statistically significant associations. Statistical analyses were performed by two-sided Fisher's exact or Chi-squared tests for categorical variables and two-sided Mann-Whitney U tests for ordinal or numerical variables. None of the clinicopathological associations was statistically significant after correcting for multiple testing. (**g**) Forest plot showing multivariate Cox proportional-hazards model of overall survival

according to the mutation status of HCC driver genes and BCLC clinical staging. n=108. Square denotes the estimated hazard ratio; whiskers extend to 95% CI. Source data are provided as a Source Data file.

Supplementary Figure 11

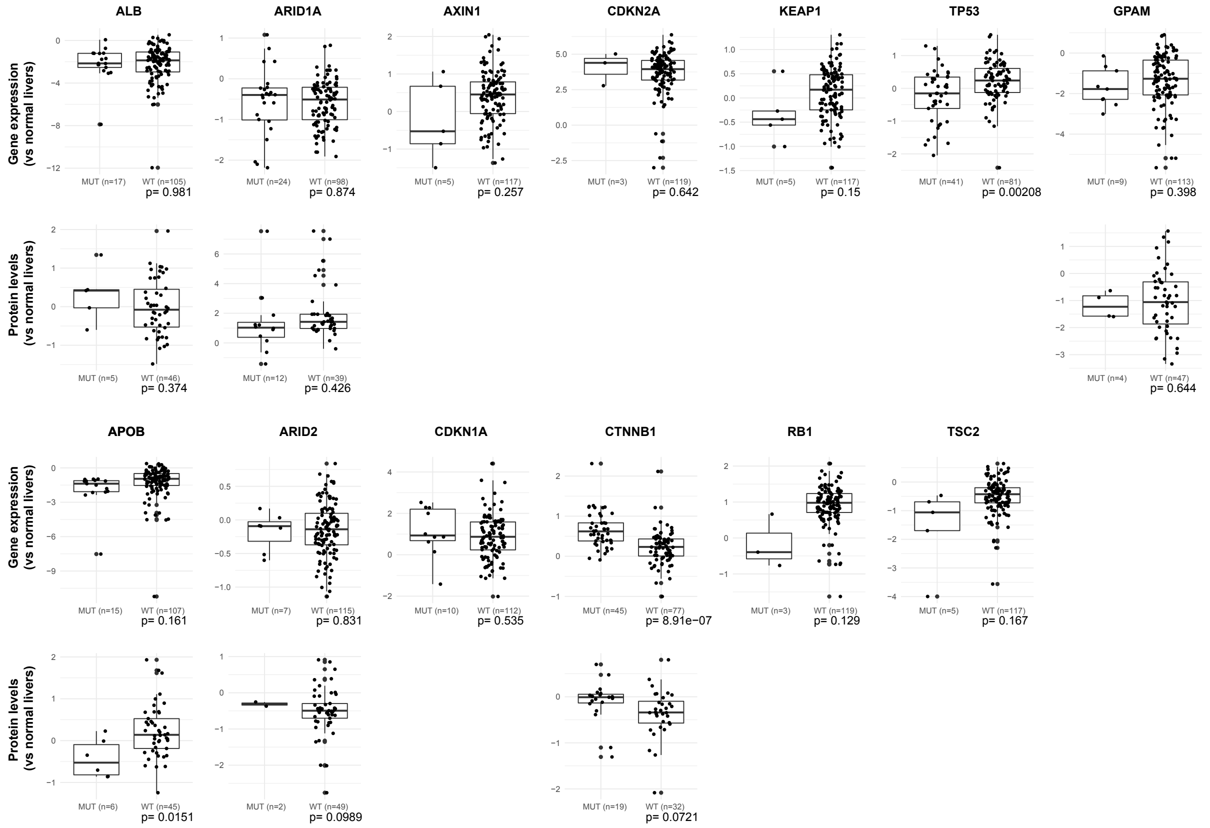

Supplementary Figure 11: The impact of the SMGs on their cognate products on the RNA and protein levels. Boxplot showing gene expression or protein levels of each of the SMGs, stratified by mutation status of the respective gene. Thick middle line in the boxplot denotes the median; box extends to the 1st and 3rd quartiles; whiskers extend to the +/-1.5 IQR of the box; dots depict the outliers. Statistical comparisons were performed by two-sided Mann-Whitney U tests. Correction for multiple testing was not performed. MUT: mutant; WT: wild-type.

Supplementary Figure 12

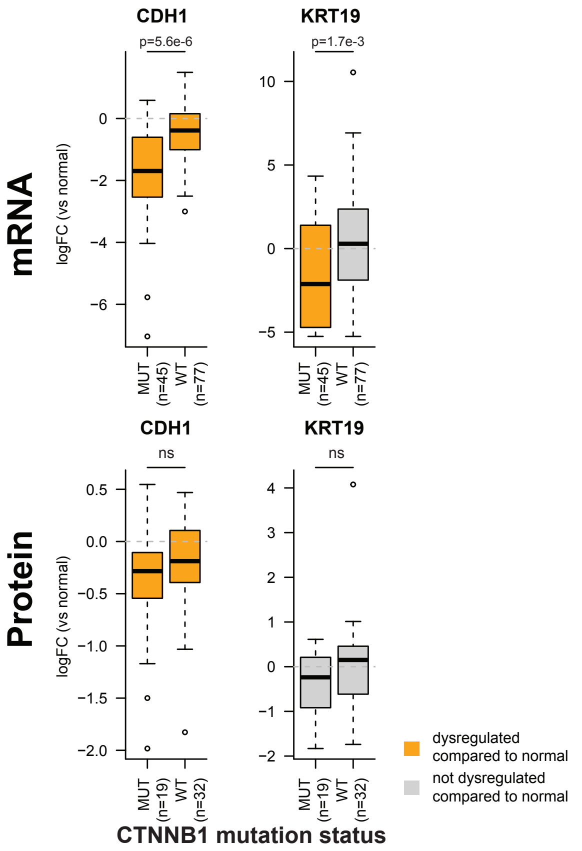

Supplementary Figure 12: Loss of epithelial phenotype in *CTNNB1*-mutant HCCs. Boxplots showing the expression of CDH1 (E-cadherin) and KRT19 (Keratin-19) in CTNNB1-mutant and -wildtype HCCs. Statistical comparisons between MUT and WT were performed using Mann-Whitney U tests. *: P<0.05. ns: not significant. Statistical comparisons between MUT and normal livers and WT and normal livers were performed using one-sample, two-sided Mann-Whitney U tests. Orange boxes indicate P<0.05. Thick middle line in the boxplot denotes the median; box extends to the 1st and 3rd quartiles; whiskers extend to the +/-1.5 IQR of the box; dots depict the outliers.

Supplementary Figure 13

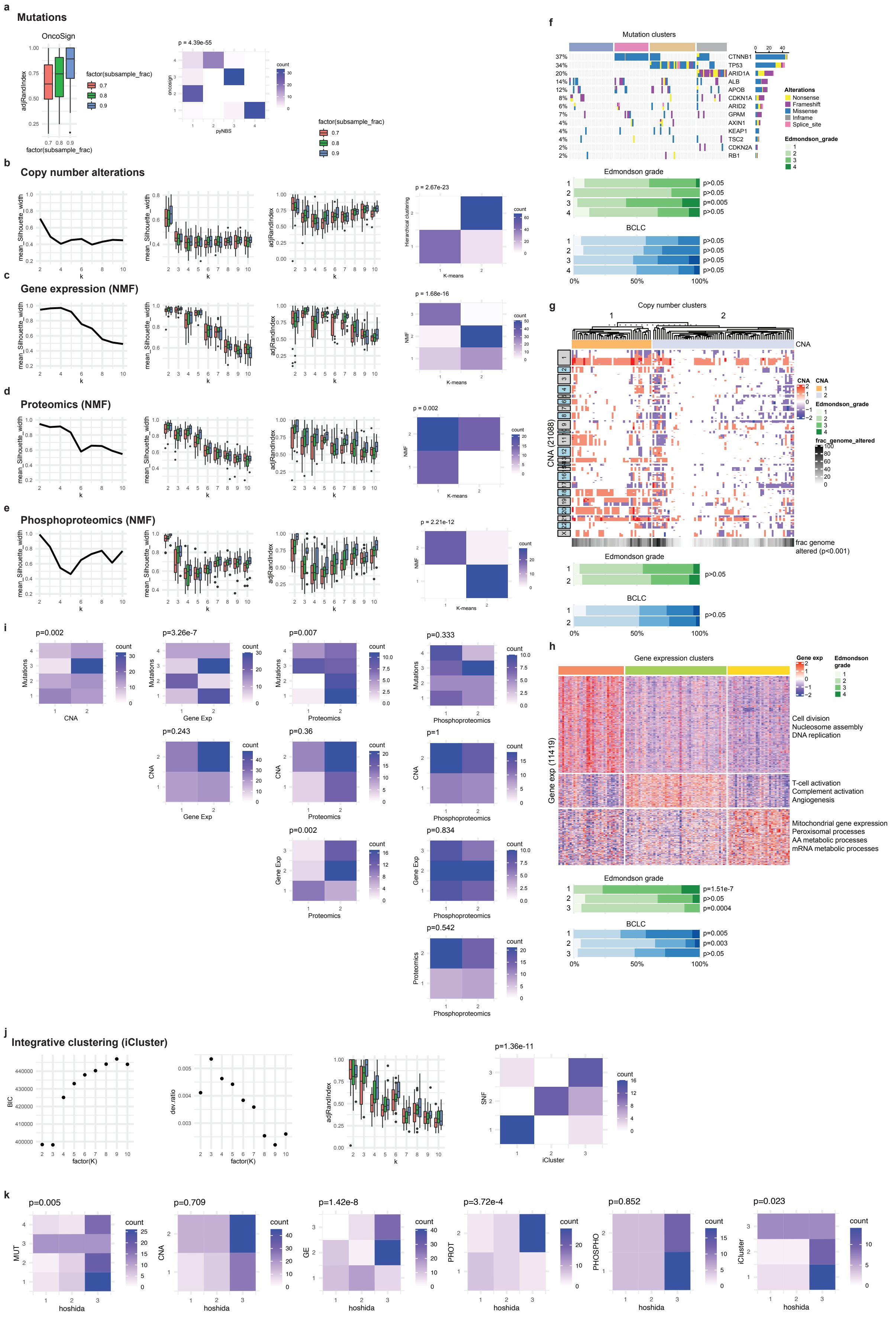

Supplementary Figure 13: For the clustering of HCC biopsies based on somatic mutations, we used OncoSign (primary) and pyNBS (alternative). For CNA, we used consensus k-means clustering (primary) and consensus hierarchical clustering (alternative). For the remaining data types, we used consensus nonnegative matrix factorization (NMF, primary) and consensus k-means clustering (alternative). We assessed the mean Silhouette width (cluster quality) and adjusted Rand index (concordance with the classes derived from the full dataset) by subsampling 70%, 80% and 90% of the samples. (**a,** left) Boxplot showing adjusted Rand index between the classes derived from OncoSign (single-omics clustering using mutation data) using the full data set and the classes derived from downsampled data. n=downsampling (70%, 80% and 90% of the samples) over 20 iterations. (right) Concordance between clusters derived from OncoSign and pyNBS. (**b-e**, from left) Mean silhouette widths against k (the number of clusters), mean silhouette widths against k from clustering using downsampled datasets (70%, 80% and 90% of the samples, n=20 iterations of downsampling), adjusted Rand index between the classes derived from the full dataset and the classes derived from subsampled data using the primary clustering method, and concordance between the primary and alternative clustering methods. Statistical comparisons were performed by two-sided Fisher's exact or chi-squared tests. **(f)** Clusters derived from OncoSign based on the significantly mutated genes found in this study and in previous studies (see methods). **(g)** Clusters derived from copy number alterations by consensus K-means clustering. **(h)** Clusters derived from gene expression by consensus NMF. (**f-h**) Barplots below show the distribution of Edmondson grade and BCLC between the clusters. Statistical comparison for each cluster was computed using the two-sided Mann-Whitney U test. **(i)** Concordance between single-omics clusters. Statistical comparisons were performed by two-sided Fisher's exact or chi-squared tests. **(j,** top**)** Assessment of the Bayesian Information Criterion and the deviance ratio (interpreted as percent explained variation) from integrative clustering using iClusterBayes. (bottom left) Boxplot showing adjusted Rank index between the classes derived from integrative clustering using the full data set and the classes derived from downsampled data. n=downsampling (70%, 80% and 90% of the samples) over 20 iterations. (bottom right) Concordance between clusters derived from iClusterBayes and SNF. Statistical comparison was performed using a chi-squared test. **(k)** Concordance between single-omics clusters and iCluster with Hoshida molecular subtypes. Statistical comparison was performed using a chi-squared test. (**a-e,j**) Thick middle line in the boxplot denotes the median; box extends to the 1st and 3rd quartiles; whiskers extend to the +/-1.5 IQR of the box; dots depict the outliers. Source data are provided as a Source Data file.

Supplementary Table

Univariate analyses					Multivariate analyses				
**Molecular cluster**	**N**	**Hazard ratio**	**pvalue**	**Global pvalue**	**Molecular cluster/ BCLC**	**N**	**Hazard ratio**	**pvalue**	**Global pvalue**
MUT1	111	1.02 (0.631-1.65)	0.938	0.938	MUT1	111	1.21 (0.746-1.98)	0.436	1.08e-09
					BCLC		2.46 (1.884-3.21)	3.59e-11	
MUT2	112	0.792 (0.475-1.32)	0.37	0.36	MUT2	112	0.534 (0.309-0.924)	**0.025**	**1.6e-10**
					BCLC		2.688 (2.005-3.605)	3.93e-11	
MUT3	111	1.41 (0.886-2.24)	0.147	0.157	MUT3	111	1.67 (1.04-2.69)	**0.034**	**2.62e-10**
					BCLC		2.50 (1.91-3.27)	2.42e-11	
MUT4	112	0.879 (0.515-1.5)	0.634	0.63	MUT4	112	0.902 (0.527-1.54)	0.707	1.58e-09
					BCLC		2.394 (1.845-3.11)	5.27e-11	
CNA1	109	1.15 (0.747-1.77)	0.526	0.524	CNA1	109	1.08 (0.702-1.67)	0.719	1.42e-09
					BCLC		2.39 (1.844-3.10)	5.30e-11	
CNA2	109	0.87 (0.565-1.34)	0.526	0.524	CNA2	109	0.924 (0.599-1.42)	0.719	1.42e-09
					BCLC		2.393 (1.844-3.10)	5.30e-11	
GE1	110	1.64 (1.03-2.62)	**0.0373**	**0.044**	GE1	110	1.53 (0.949-2.45)	0.081	6.85e-09
					BCLC		2.28 (1.742-2.98)	1.8e-09	
GE2	110	0.719 (0.465-1.11)	0.138	0.135	GE2	110	0.832 (0.533-1.30)	0.42	1.23e-08
					BCLC		2.301 (1.758-3.01)	1.27e-09	
GE3	111	0.961 (0.595-1.55)	0.872	0.872	GE3	111	0.838 (0.516-1.36)	0.475	2.01e-09
					BCLC		2.410 (1.851-3.14)	6.84e-11	
PROT1	49	2.23 (1.12-4.41)	**0.0218**	**0.027**	PROT1	49	1.46 (0.699-3.03)	0.316	3.17e-09
					BCLC		5.14 (2.893-9.11)	2.28e-08	
PROT2	49	0.449 (0.227-0.89)	**0.0218**	**0.027**	PROT2	49	0.687 (0.33-1.43)	0.316	3.17e-09
					BCLC		5.135 (2.89-9.11)	2.28e-08	
PHOSPHO1	47	0.935 (0.478-1.83)	0.845	0.845	PHOSPHO1	47	0.774 (0.387-1.55)	0.468	9.07e-09
					BCLC		5.419 (3.062-9.59)	6.60e-09	
PHOSPHO2	47	1.07 (0.547-2.09)	0.845	0.845	PHOSPHO2	47	1.29 (0.646-2.58)	0.468	9.07e-09
					BCLC		5.42 (3.062-9.59)	6.60e-09	
ICLUSTER1	49	1.03 (0.528-2.03)	0.921	0.921	ICLUSTER1	49	1.30 (0.632-2.68)	0.475	4.01e-09
					BCLC		5.64 (3.197-9.94)	2.31e-09	
ICLUSTER2	48	0.304 (0.117-0.791)	**0.0147**	**0.00561**	ICLUSTER2	48	0.551 (0.20-1.51)	0.248	4.09e-09
					BCLC		4.823 (2.71-8.58)	8.52e-08	
ICLUSTER3	48	2.62 (1.33-5.13)	**0.00515**	**0.00625**	ICLUSTER3	48	1.24 (0.542-2.82)	0.613	7.54e-09
					BCLC		5.01 (2.721-9.23)	2.29e-07	

Supplementary Table 1: Univariate and multivariate Cox proportional hazards analyses for each single-omics and integrative clusters. Related to Figure 5 and Supplementary Figure 13.
