# Supplementary material for: Integrative proteogenomic characterization of hepatocellular carcinoma across etiologies and stages
Source: Nat Commun. 2022 May 4;13:2436. doi: 10.1038/s41467-022-29960-8 (PMC9068765; doi:10.1038/s41467-022-29960-8)
Supplement: Supplementary file 3 — Description of Additional Supplementary Files [file 41467_2022_29960_MOESM3_ESM.pdf]

Supplementary Data legends

Supplementary Data 1: Clinicopathological parameters of the cohort, as well as technical details of molecular profiling. Related to Table 1.

Supplementary Data 2: List of enriched Reacome, KEGG and Gene Ontology biological processes for deregulated pathways on the mRNA and protein levels. Enrichment was assessed by over-representation tests. Correction for multiple comparison was performed using the Benjamini-Hochberg method. Related to **Figure 1f**.

Supplementary Data 3: List of enriched Reacome, KEGG and Gene Ontology biological processes for genes and proteins differentially expressed between BCLC B/C/D vs A/0 HCCs. Enrichment was assessed by gene set enrichment analysis. Correction for multiple comparison was performed using the Benjamini-Hochberg method. Related to **Supplementary Figure 7b-c**.

Supplementary Data 4: List of enriched Reactome/KEGG pathways/GISTIC2 peaks among genes with high CNA-mRNA expression correlation and genes with high mRNA-protein expression correlation. Enrichment was assessed by gene set enrichment analysis. Correction for multiple comparison was performed using the Benjamini-Hochberg method. Related to Figure 2c-d and Supplementary Figure 8.

Supplementary Data 5: List of genes with high (rho≥0.5) CNA-mRNA and mRNA-protein correlations. Genes within GISTIC2 peaks and GISTIC2 peaks enriched among genes with high CNA-mRNA correlation are annotated. Genes enriched among GISTIC peaks were further evaluated for dysregulation (relative to normal livers). Correlations were assessed by the Spearman correlation test. Correction for multiple comparison was performed using the Benjamini-Hochberg method. Related to Figure 2d.

Supplementary Data 6: List of dysregulated phosphosites in HCC. Differential expression analysis was performed using the limma package. Correction for multiple comparison was performed using the Benjamini-Hochberg method. Related to Figure 3a and Supplementary Figure 3a.

Supplementary Data 7: List of enriched Reacome, KEGG and Gene Ontology biological processes for deregulated pathways on the phosphoprotein levels. Enrichment was assessed by gene set enrichment analysis. Correction for multiple comparison was performed using the Benjamini-Hochberg method Related to Figure 3b and Supplementary Figure 3b.

Supplementary Data 8: List of kinases with altered activity in HCC and their associated substrates (at least a 5-fold difference between HCCs and normal livers). Analysis was performed using a Kinase-Substrate Enrichment Analysis (KSEA). Correction for multiple comparison was performed using the Benjamini-Hochberg method. Related to Figure 3c and Supplementary Figure 3c.

Supplementary Data 9: List of somatic mutations. Related to Figure 4 and Supplementary Figure 10a.

Supplementary Data 10: Association between mutation status and clinicopathological parameters. Statistical analyses were performed by two-sided Fisher's exact or Chi-squared tests for categorical variables and two-sided Mann-Whitney U tests for ordinal or numerical variables. No correction for multiple comparison was performed. Related to Supplementary Figure 10f.

Supplementary Data 11: List of proteins differentially expressed between *CTNNB1*/*TP53*-mutant vs wild-type HCCs and their corresponding differential expression on the mRNA level. Differential expression analysis was performed using the edgeR (mRNA) or the limma (protein) package. Correction for multiple comparison was performed using the Benjamini-Hochberg method. Related to Figure 4a/e.

Supplementary Data 12: List of Gene Ontology biological processes enriched among proteins with phosphorylation sites at >90th quantile of the unsigned p-values of the differential phosphoprotein expression analysis and within the inter-quartile range of signed p-values of differential protein expression analysis for *CTNNB1*- and *TP53*- mutant HCC. Enrichment was assessed by over-representation tests. Correction for multiple comparison was performed using the Benjamini-Hochberg method. Related to Figure 4c/g.

Supplementary Data 13: Pathway/KSEA analysis for single-omics subclasses. Enrichment was assessed by over-representation tests (pathways) or Kinase-Substrate Enrichment Analysis (KSEA). Correction for multiple comparison was performed using the Benjamini-Hochberg method. Related to Figure 5a-b and Supplementary Figure 13h.

Supplementary Data 14: Pathway/KSEA analysis for integrative clustering (iCluster) subclasses. Enrichment was assessed by over-representation tests (pathways) or Kinase-Substrate Enrichment Analysis (KSEA). Correction for multiple comparison was performed using the Benjamini-Hochberg method. Related to Figure 5c.
